# RETRACTION: A Review on Wound Management Strategies in Enhanced Recovery After Craniotomy: An In‐Depth Analysis of their Influence on Patient Recovery and Surgical Outcomes

**DOI:** 10.1111/iwj.70489

**Published:** 2025-04-02

## Abstract

**RETRACTION:** WuN. and LiL. , “A Review on Wound Management Strategies in Enhanced Recovery After Craniotomy: An In‐Depth Analysis of their Influence on Patient Recovery and Surgical Outcomes,” International Wound Journal 21, no. 1 (2024): e14595, 10.1111/iwj.14595.38272808 PMC10789584

The above article, published online on 15 January 2024, in Wiley Online Library (http://onlinelibrary.wiley.com/), has been retracted by agreement between the journal Editor in Chief, Professor Keith Harding; and John Wiley & Sons Ltd. Following an investigation by the publisher, all parties have concluded that this article was accepted solely on the basis of a compromised peer review process. The editors have therefore decided to retract the article. The authors did not respond to our notice regarding the retraction.

**RETRACTION:** N. Wu and L. Li , “A Review on Wound Management Strategies in Enhanced Recovery After Craniotomy: An In‐Depth Analysis of their Influence on Patient Recovery and Surgical Outcomes,” International Wound Journal 21, no. 1 (2024): e14595, 10.1111/iwj.14595.38272808 PMC10789584

The above article, published online on 15 January 2024, in Wiley Online Library (http://onlinelibrary.wiley.com/), has been retracted by agreement between the journal Editor in Chief, Professor Keith Harding; and John Wiley & Sons Ltd. Following an investigation by the publisher, all parties have concluded that this article was accepted solely on the basis of a compromised peer review process. The editors have therefore decided to retract the article. The authors did not respond to our notice regarding the retraction.

